# Oral healthcare for older adults in Swedish municipal healthcare—a qualitative study of healthcare professionals’ experiences

**DOI:** 10.1186/s12877-025-05764-5

**Published:** 2025-02-18

**Authors:** Maria Snogren, Kristina Ek, Ulrika Lindmark, Maria Browall, Irene Eriksson

**Affiliations:** 1 School of Health Sciences, University of Skövde, Box 408, Högskolevägen, Skövde 541 28 Sweden; 2 Research School of Health and Welfare, Jönköping University, Jönköping, Sweden; 3 The Faculty of Health, Science and Technology, Department of Health Sciences, Karlstad University, Karlstad, Sweden; 4 Department of Nursing, School of Health and Welfare, Jönköping Academy for Improvement of Health and Welfare, Jönköping University, Jönköping, Sweden; 5 Faculty of Caring Science, Work Life and Social Welfare Department of Caring Sciences, University of Borås, Borås, Sweden; 6 Department of Oncology, Institute of Clinical Sciences, Sahlgrenska Academy, University of Gothenburg, Gothenburg, Sweden; 7 Department of Health Sciences, University West, Trollhättan, Sweden

**Keywords:** Fundamentals of care, Healthcare professionals, Municipal healthcare, Oral healthcare

## Abstract

**Introduction:**

Oral health is multi-faceted and influences a person’s daily life, and numerous potential barriers and factors can challenge and pose barriers to good oral health. Shortages of healthcare professionals or incorrect care practices can be barriers to performing good oral healthcare. A knowledge gap has been identified in qualitative research on description of healthcare professionals’ experiences of oral healthcare among older adults in municipal healthcare.

**Aim:**

To describe healthcare professionals’ experiences of oral healthcare among older adults in Swedish municipal healthcare.

**Design and methods:**

The study employed a qualitative design guided by a secondary qualitative analysis method comprising inductive qualitative content analysis. Data were collected through semi-structured individual interviews with healthcare professionals.

**Results:**

Good relationships and mutual trust create the conditions for delivering good oral healthcare. Knowledge provides confidence and trust in performing oral healthcare, experiences and strategies influence the individual adaptation of oral healthcare, and priorities and collaboration influence oral healthcare provision.

**Conclusion and implications:**

The performance of oral healthcare is complex and cannot be achieved without establishing a relationship with the older adult who needs care. Oral healthcare includes prerequisites such as routines, sufficient time, work-time planning, and collaboration between healthcare professionals. Registered nurses positively experience opportunities to collaborate with other healthcare professionals regarding oral healthcare and are seen as leaders in the Fundamentals of Care and are sharing good examples of oral healthcare in palliative care.

## Introduction

As for many other countries the Swedish population is rapidly ageing [1, 2], which contributes to older adults with extensive and complex care needs such as oral healthcare in municipal healthcare [3]. Municipal healthcare includes healthcare in the individual’s home (home healthcare), nursing home care, or short-term care. ‘Healthcare’ refers to the efforts needed to meet physical, psychological, and social needs. This can, for example, mean help with eating and drinking, dressing, and moving around, as well as taking care of personal hygiene such as oral healthcare [4].

The increasing proportion of older adults in society [1, 2], most of whom have their own teeth intact or have advanced implants, has an effect on oral healthcare provision [5]. Advanced implants entail a need for more complex oral healthcare to maintain good oral health for older adults which includes the need for, among other things, training among healthcare professionals (registered nurses, assistant nurses, and care assistants) and older adults [1, 5]. Oral healthcare ensures quality of care and contributes to patient safety [6, 7]. Oral healthcare improves oral hygiene, oral function, ingestion, and swallowing ability to support nutrition, hydration, and speech and thereby improves general health [6, 8]. Altogether, improved oral health can support older adults (a person aged 65 years or older) in need of care to live more independently and helps reduce their need for care [9]. Oral health is related to social and behavioural factors such as socioeconomic status and health habits [5]. Poor oral health can be prevented among older adults in municipal healthcare when oral healthcare is provided by healthcare professionals [10, 11]. Registered nurses are responsible for promoting the Fundamentals of Care (FoC), such as oral healthcare, and for ensuring that it is carried out and that information about oral healthcare reaches other healthcare personnel who perform it [12, 13]. The FoC are defined in the FoC framework as being essential elements of care performed by healthcare professionals that respect and focus on a person’s basic needs to ensure their physical and psychosocial well-being, regardless of disease, illness, or the environment in which care is provided [14]. The FoC framework describes how important it is for healthcare professionals to have knowledge that can be adapted to individual clinical care settings to achieve good FoC. The framework also describes the importance of moving care from a series of tasks to coordinated, integrated, person-centred care, such as oral healthcare [14, 15]. Oral healthcare is complex, and includes barriers such as lack of time, lack of knowledge, attitudes towards performing oral healthcare, or feelings of unpleasantness in performing oral healthcare, and perceptions that it is a violation of dignity [16–18]. Shortages of healthcare professionals or incorrect care practices can also be barriers to performing oral healthcare. Oral healthcare is a commonly de-prioritised element of care among healthcare professionals [7]. However, research has found that registered nurses in municipal healthcare report being aware of the impact of oral health on overall health. Still, oral healthcare is considered private and can be challenging to provide if the older adult resists it [17]. Lack of preparedness for unexpected situations, obstacles in a deficient work environment, unsatisfactory planning within the organisation, and/or shortcomings related to the individual all affect the provision of oral healthcare [6, 7]. A lack of resources can also present barriers to oral healthcare. Sufficient supplies, products [19], routines, and guidelines [20] are crucial for healthcare professionals in performing oral healthcare. The most frequently mentioned barrier to performing oral healthcare in previous research relates to older adults’ behaviour and unwillingness to cooperate, often caused by cognitive impairments such as dementia [19, 21].

In summary, performing oral healthcare is complex, and depends on what is considered private [17], supplies and products [19], routines, guidelines [20], and behavioural factors among older adults [19, 21].

Since the population is living longer, which contributes to a higher proportion of older adults in the population with care needs such as oral healthcare, which places demands on the resources and competence of healthcare professionals [2, 6]. Oral health is also considered a sensitive marker for the population’s health and a global health challenge [4]. Previous research has focused on older adults’ oral health status [8], healthcare professionals’ inspection/assessments [22], and instruments to measure older adults’ oral health status [23], each with a quantitative research focus, and only a limited number have focused on healthcare professionals’ experience [24]. Therefore, a knowledge gap exists in qualitative research on the description of healthcare professionals’ experiences of oral healthcare among older adults in municipal healthcare. Qualitative research focusing on nurses’ perspectives on oral healthcare can contribute to a deeper understanding of the complexities and potential limitations of existing research and knowledge of oral healthcare. Therefore, the aim was to describe healthcare professionals’ experience of oral healthcare among older adults in Swedish municipal healthcare.

## Methods

### Design

The study employed a qualitative design guided by a secondary qualitative method [25] to describe healthcare professionals’ experiences of oral healthcare among older adults in Swedish municipal healthcare. Data were collected through semi-structured individual interviews with healthcare professionals, which were then analysed using inductive qualitative content analysis [26, 27]. COnsolidated criteria for REporting Qualitative research (COREQ) [28] was taken into consideration when conducting the study.

### Participants and recruitment

The sample was comprised of healthcare professionals (registered nurses, nursing assistants, and care assistants) in municipal healthcare (nursing homes, short-term care, and homecare) in an urban area in western Sweden with a total number of 94 permanently employed healthcare professionals. The municipality is responsible for providing nursing care for in-home healthcare services and municipality-run nursing homes. Participants were recruited by providing written and verbal information about the study to the municipality’s head of healthcare services, who identified and asked informants to participate. The inclusion criteria were: permanently employed healthcare professionals who can read and understand Swedish and who had undergone an intervention with a digital training module in oral health in spring 2022 which has been described in an earlier publication by Snogren, Ek [29]. Semi-structured individual interviews with healthcare professionals were conducted in the spring of 2023, and the interviews included two registered nurses, thirteen assistant nurses, and one care assistant. One participant was a man, and fifteen were women. The length of professional experience ranged from two to forty years, with a median of seventeen years. Three worked in home healthcare, ten in nursing homes, and three in short-term care.

### Data collection

The semi-structured interview questions were based on previously completed focus groups with nine healthcare professionals [30] performed in spring 2022 before they participated in an intervention that included a digital training module in oral health [29]. The aim of the focus groups was to create a starting point and inspiration for the semi-structured individual interviews [25]. The questions, based on the focus group discussions, covered knowledge about oral health, performing oral healthcare, and prerequisites for oral healthcare, and all authors were involved in the design of the questions. All individual semi-structured interviews opened with the same question: “Can you describe what promotes good oral health?”, which was followed by questions focusing on, for example, “Can you describe a situation where you felt that you were able to perform oral healthcare in a good way?”, and “Can you describe which conditions can affect the performance of oral healthcare?” These were followed by questions covering the informant’s skills, information-seeking activities, and prerequisites and ethics related to oral healthcare. Subsequent follow-up questions were used based on each informant’s response and orientation in the dialogue to deepen understanding. Background data, such as each participant’s gender, profession, years in the profession, and workplace, were collected. The interviews were performed remotely, using digital video conferencing software, and the head of healthcare services arranged a suitable place and time for the healthcare professionals to participate and were conducted by the same person (MS) (lasting between 9 and 30 min with an average length of 24 min). The participants were able to ask questions and obtain information about the study, and were asked to provide consent to participate before the interviews began. All interviews were audio-recorded using the video software function and supporting notes were taken during each interview. All interviews were transcribed verbatim and manually analysed by all authors.

### Data analysis

The data from the semi-structured individual interviews were analysed using inductive qualitative content analysis [26, 27] guided by a secondary qualitative data analysis method [25]. The analysis included five steps, and was an interactive process that moved back and forth across the steps, not linearly [26, 27]. The interview text was read several times in the first step to obtain a feel for the whole. Text that was determined to be significant to the aim of the research was then extracted and brought together into one text, constituting the analysis unit in a second step. In the third step, meaning units related to the purpose were identified and condensed, where the meaning of the text was preserved. In the fourth step, the condensed meaning units were abstracted and labelled with a code. Context was taken into account when condensing and coding. Codes that highlighted the meaning of the condensation were created. The codes were compared based on differences and similarities. In the fifth step, the codes were sorted into categories which reflected the data’s manifest content. The common content was presented in a main overarching category [26, 27]. The analysis resulted in an overarching category and three subcategories. All authors read the interview texts several times and discussed and reflected upon them together. To secure trustworthiness, the interviews were analysed manually separately, then the resulting categories and subcategories were compared and discussed by all authors to reach a consensus [26, 27].

### Etichal considerations

The study was conducted in accordance with principles outlined in the Declaration of Helsinki [31], and was approved by the Swedish Ethical Review Authority in Umeå (Dnr: 2020–02755). Participating healthcare professionals were informed that their participation was voluntary and that they could withdraw at any time without stating a reason. The participants signed written informed consent forms. The transcribed interviews were analysed after being anonymised by replacing identifying information with pseudonyms.

## Results

The qualitative content analysis of the data material resulted in an overarching category: *good relationships and mutual trust create the conditions for delivering good oral healthcare*, and three subcategories: *knowledge provides confidence and trust in performing oral healthcare*, *experiences and strategies influence individual adaptation of oral healthcare*, and *priorities and collaboration influence oral healthcare provision*. The overarching category and subcategories are described in detail below, and quotations from the interviews are used to exemplify the results.

### Good relationships and mutual trust create the conditions for delivering good oral healthcare

Good relationships and mutual trust permeated the results as a whole and were seen as an essential component when the healthcare professionals provided oral healthcare. Maintaining a good relationship in the provision of oral healthcare necessitates well-established and mutual trust between the healthcare professionals and the older adult in question. Oral healthcare was considered complex and multidimensional, and trust was established through good relationships in the provision of oral healthcare. Good availability of healthcare professionals, continuity, routines, collaboration, and sufficient time provided opportunities to develop mutual trust. Mutual trust was perceived as being challenging to obtain when the organisation discourages relationship-building and there is a lack of participation from registered nurses in daily care. Creating the conditions for delivering good oral healthcare was also described as occurring through small actions enacted by healthcare professionals, such as making eye contact with the older adults, which creates good relationships and mutual trust.

### Knowledge provides confidence and trust in performing oral healthcare

Having knowledge of the older adult was described as facilitating the provision of oral healthcare, and getting to know older adults could take time but provided reassurance. Oral healthcare was described as being impossible without first establishing a relationship and building knowledge about the older adult who needed help. The relationship was mainly created through conversation and provided security and trust. An assistant nurse described:

The relationship means that you create something—what can I say—a security that I know that I get good support from you and that you are a good person who does not want to [harm me]. You wish me well, so to speak

Healthcare professionals experienced their work as being rewarding when they developed relationships with and knowledge of older adults and when care was provided based on the older adults’ needs and wishes. Task-focused care, where oral healthcare was prioritised over the relationship, was described as creating obstacles to developing confidence in their knowledge about the older adult.

Engaging in continuing professional development for healthcare professionals in oral health and oral healthcare, along with updating their knowledge, facilitated and led to confidence in providing oral healthcare. However, the importance of care assistants, assistant nurses, registered nurses, and other healthcare professionals receiving education together was also described. Receiving education together was perceived to promote confidence in providing care and opportunities to share knowledge among each other. Registered nurses were described as having the opportunity to fulfil the function of educating assistant nurses and care assistants in their daily work, such as assessing and examining oral healthcare needs. The healthcare professionals also described physiotherapists and occupational therapists as having knowledge of assistive devices and training concerning oral healthcare. Assistant nurses and care assistants were described as the individuals who performed oral healthcare daily and who had the most significant knowledge of how oral healthcare was provided and documented. Care assistants and assistant nurses described the benefit of creating developmental opportunities for collaboration with registered nurses, physiotherapists, and occupational therapists, as they were seen to have expertise in their respective professions. Continuity in providing oral healthcare was assured when healthcare professionals shared information verbally and documented tips and techniques for providing oral healthcare in the care record. Detailed documentation that included information about dentures; the number of teeth, dental bridges, crowns, and implants; the appearance of the mucosa; any dental fear; and a detailed description of provision preferences facilitated and led to confidence in the provision of oral healthcare. An assistant nurse described this as:

But if you look in the mouth, you want to know what you see and what is right for this person

Healthcare professionals described how knowing the older adult’s life story is crucial for creating an understanding about the older adult and considered it essential to document this. The life story was either obtained from the older adult or their next-of-kin, which could provide pieces to a life puzzle that were added to over time. Next-of-kin were described as an essential source of information when older adults could not describe their life stories themselves. Documentation from other healthcare providers, such as primary healthcare, was often perceived as being inadequate regarding oral health and oral healthcare. However, documentation on oral health and oral healthcare was more likely to be recorded if the older adult had had a neurological disease, such as a stroke, or if the documentation came from dental healthcare.

### Experiences and strategies influence individual adaptation of oral healthcare

Healthcare professionals’ own experiences of oral health and oral healthcare were described as influencing strategies for providing oral healthcare. Healthcare professionals’ own dental fear could lead to uneasiness in providing oral healthcare to older adults. Fears in providing such care could lead to insecurity, as the older adults’ dignity was affected. Dignity was perceived as compromised when in close proximity to the older adult’s face when providing oral healthcare. However, satisfaction was experienced in helping someone who needed help, as one assistant nurse described:

I think it’s more personal; some people find it easier to perform oral healthcare than others, despite affecting the older adults’ dignity

The provision of oral healthcare was promoted through personalisation, with healthcare professionals asking questions about habits and routines and offering assistance on several occasions. Providing opportunities to perform oral healthcare in stages, such as helping older adults rinse their mouth after a meal, wipe their mouth, and use a toothbrush, were seen as ways of providing oral healthcare. Likewise, providing information while maintaining eye contact and adapting the provision to the older adults’ needs was essential. Adapting healthcare professionals’ practices was a strategy adopted when a healthcare professional did not manage to provide oral healthcare. Strategies such as asking questions again at a later time or using fluoride tablets as a complement to oral healthcare were described as adapting healthcare professionals’ practices in providing oral healthcare. Healthcare professionals also used multiple strategies to individualise the provision of oral healthcare. One example was to let the older adult provide oral healthcare according to their ability, and healthcare professionals would assist afterwards. However, brushing a few teeth, or simply rinsing the mouth or using fluoride tablets as a supplement could be sufficient. Allowing the older adult to feel and smell the toothbrush and toothpaste and using two toothbrushes—one for the healthcare professionals and one for the older adult—were also described as strategies for providing good oral healthcare. Mirroring and imitating what the older adult should do also promoted the provision of oral healthcare when asking the older adult to open their mouth. For example, healthcare professionals opening their mouths, which could lead to older adults opening their mouths. Oral healthcare was then joked about through mimicry and conversation. For older adults affected by cognitive impairment, healthcare professionals described that statements were more accessible and easier for them to interpret than questions, as one assistant nurse described:

I usually just say something like “Let’s go to bed, and then we’ll brush our teeth” or “We’ve had breakfast, so now we’ll brush our teeth” or something like that because if you ask, it’s usually a no…

Physical changes such as weakness in one half of the body after a stroke or side effects of medication were described as affecting how oral healthcare was provided. Assistive devices, such as toothbrushes with colours, different sizes, or other forms of assistive devices, facilitated and individualised how oral healthcare was performed, for example, after a stroke.

### Priorities and collaboration influenced oral healthcare provision

Healthcare professionals’ priorities in relation to older adults’ priorities influenced the performance of oral healthcare, with one assistant nurse describing it as follows:

Many healthcare professionals are more careful about washing genitalia than about taking care of older adults’ oral healthcare

Oral healthcare was de-prioritised when other care actions needed to be prioritised and when there was a lack of time. Having to tick oral healthcare on a checklist to show that it had been performed was described as promoting that oral healthcare had been prioritised and provided. An improvement was also described where a checklist was supplemented with questions asking the older adults about their habits, experiences, and wishes regarding oral healthcare to gain an understanding of these as healthcare professionals. Establishing clear and informative routines for healthcare professionals on what oral healthcare includes was seen as an additional improvement. Access to a toothbrush and toothpaste, which were assumed to be available, was described as being fundamental to performing oral healthcare. Therefore, supporting older adults in purchasing a toothbrush and toothpaste was described as a primary necessity. Next-of-kin could also provide support if any purchases were necessary. However, access to time and routines was also a significant factor influencing oral healthcare.

Continuity in how and when oral healthcare was provided was described as being essential and was promoted through good continuity among healthcare professionals. Continuity of care between healthcare professionals was influenced by shift scheduling and handover by healthcare professionals to the next shift. Many daily issues were resolved by talking to a colleague. Oral healthcare was seen as a shared responsibility between different healthcare professions. However, assistant nurses and care assistants were described as taking great responsibility for oral healthcare and they consulted with registered nurses, physiotherapists, and occupational therapists only when necessary. Assistant nurses and care assistants described a desire to collaborate further with registered nurses, physiotherapists, and occupational therapists. Registered nurses described positive experiences of working together and being involved with assistant nurses and care assistants in planning the care design, as risk assessments and preventive measures could be carried out continuously. One registered nurse described it as follows:

You would like to have more control and be more involved in the care. You see a lot more, discover things like how they are, and how they walk and eat and take care of themselves, take their medication

The optimal standard of collaboration was described in palliative care. In this setting, the registered nurse was present in the daily care routines and supported and educated the assistant nurses and care assistants. These were described as promoting the performance of oral healthcare. Healthcare professionals described having opportunities to collaborate with other professional disciplines (such as occupational therapists and physiotherapists) regarding aids or training that could promote the provision of oral healthcare. Healthcare professionals experienced a lack of collaboration on oral healthcare with care coordinators, as they rarely considered oral health and oral healthcare when investigating older adults’ need for care. Visits with dental professionals in older adults’ homes promoted interaction between healthcare professionals and dental professionals. This contributed to more healthcare professionals being present and receiving direct oral and written information from dental healthcare professionals on how oral healthcare should be provided. The examination of oral health by dental healthcare professionals at home was also described as promoting oral healthcare over time. However, visiting a dental healthcare clinic could become an excursion for the older adult and an interruption to everyday life when allowed by the older adults.

## Discussion

The results show that oral healthcare is complex and requires the effective integration of knowledge, strategies, collaboration, and priorities. The results also describe how valuable a good relationship between healthcare professionals and older adults is for the performance of oral healthcare.

A good relationship comprises healthcare professionals knowing the older adults’ experience of reality, significant life events, characteristics, and habits in order to perform good oral healthcare.

A good relationship in healthcare requires understanding older adults’ experiences and habits to provide effective oral care, which fosters trust and positivity and develops a positive and trusting relationship. Establishing a relationship can take time, but is created when healthcare professionals are present, anticipate needs, have good knowledge, and evaluate and reflect [12]. Being provided with sufficient time to establish a relationship is described in the results of the present study as a critical factor in the performance of good oral healthcare, and leads to the expansion of professional knowledge, which also is described in relation to the FoC framework [12]. Establishing good relationships and taking the time to learn about older adults’ life stories helps in gaining knowledge and understanding of the older adults’ individual needs and wishes and an understanding of the older adults as whole individuals, which is in accordance with the ethos of person-centred care and aligns with previous research [12, 15]. Knowing older adults’ life stories also transforms care from a series of tasks to coordinated, integrated, person-centred care, which previous research describes as being in line with the FoC framework [14]. Moving oral healthcare from task-based to person-centred care also gives healthcare professionals knowledge of oral health’s impact on general health [17]. Still, oral healthcare was described in the result as a private act and could be challenging to provide if the older adult resists care, as it can be perceived as a violation of their dignity, as has also been described in previous research [16–18]. Older adults’ life stories can provide healthcare professionals with knowledge supporting the dignity of older adults. Healthcare professionals describe that the life story can reveal information about the older adults’ habits, such as a wish to rinse the mouth with water after a meal to counteract inflammation and infections in the mouth, as previously been described [10]. Rinsing the mouth is described to improve oral hygiene and oral function, helping to maintain ingestion and swallowing abilities that support nutrition and hydration, speech, and the general condition of health, and thus improve overall health [8].

Establishing a relationship was described in the result as also supporting healthcare professionals in learning how questions and statements are crucial; for example, clear directives such as “now we are going to brush our teeth” can be considered more accessible for older adults compared to asking open-ended questions about oral healthcare. Older adults’ cognitive ability could be an obstacle to the healthcare professionals’ interpretation and understanding of the situation when the older person declines oral care, which has also been described in previous research [9]. Establishing a relationship was described in the result as a process that leads to knowledge and reflection. Reflection and conversation allow healthcare professionals to discuss different courses of action based on theoretical and practical knowledge, as well as previous experiences, and gain understanding and knowledge of the situation which should be prioritised by healthcare personnel and facilitated within the healthcare organisation [24]. Strategies such as asking questions again at a later point, using fluoride tablets, or changing professional practices were also described in this study as preventing poor oral health, as has been previously described [22].

Healthcare professionals need education and proper training to perform adequate inspections and assessments of oral health [22]. Our study’s results indicate that education is essential in reducing difficulties in performing oral healthcare, as also described by previous research [19]. Education was also described as significant when several healthcare professionals, such as registered nurses, assistant nurses, and care assistants attending together, a condition that are describe as being essential [24, 32]. The need for education, routines, work-time planning, and collaboration between healthcare professionals was described as being the healthcare organisation’s responsibility. In addition, the characteristics of the organisation were described as having a significant impact on the conditions for performing oral healthcare. Registered nurses perceive the organisation as being responsible for facilitating opportunities for collaboration between healthcare professionals, such as registered nurses and assistant nurses. The results also indicate that the organisation does not see registered nurses as the individuals who should perform oral healthcare. Registered nurses described how the organisation promotes the idea that they should act as a consultant for informing other healthcare professionals in performing oral healthcare, rather than being involved in daily care routines. The registered nurses, however, experience that oral healthcare works best when they are able to act both as a resource for consultation and be closely involved in collaborating with other healthcare professionals to provide good care. Previous research describes how fewer patient injuries occur in municipal healthcare when registered nurses are involved in daily care [7], and that their collaboration. is needed to provide high-quality Fundamentals of Care [15]. The study’s results revealed that good routines, strong collaboration, and organisation around oral healthcare established within palliative care settings work best when registered nurses, assistant nurses, and care assistants work closely together. Established routines, such as being able to sign off oral healthcare on a checklist to show that it has been performed were also described in the result. However, routines and signatures should only be used as guidelines, as each situation must always be considered from its unique perspective when aiming to promote good oral healthcare [19]. Collaboration between healthcare professionals and dental healthcare clinics was also described as promoting good oral healthcare. Opportunities were seen when dental healthcare clinic visits occurred in the older adults’ homes, and where older adults did not have to go to the dental healthcare clinic. However, visits to the dental healthcare clinic can also become a welcome interruption and an excursion for older adults in their everyday lives. Establishing collaborations between organisations, healthcare professionals, and dental healthcare clinics was seen as a way of developing oral healthcare in the future with shared training and knowledge exchange in a standardised way, as previous studies [19, 33, 34] have proposed as being essential to further developing effective oral healthcare practices.

### Strengths and limitations

This study’s main strength is that it explores a rarely studied area of healthcare professionals’ experiences in oral healthcare among older adults in municipal healthcare. The results can broaden understanding of the importance of oral healthcare knowledge and the provision of good person-centred oral healthcare based on relationships. The first author’s experience as a geriatric specialist nurse allowed a reflection, complemented by an awareness of healthcare professionals’ situations, and provided an opportunity for reflective discussions with all authors. Therefore, the first author made a great effort to be aware of and reflect upon the experience as a geriatric specialist nurse throughout the process. The same author carried out all the individual interviews and the focus group interviews, and the same questions were asked in the same way by the same person and by pre-testing the interview guide to strengthen the study’s credibility. Transferability were enhanced when the same questions were asked in the same way by the same persons. Authenticity was secured by including original quotations when describing the results. Credibility was also confirmed by including all authors in the analysis process.

The study comprised a number of different healthcare professionals working in municipal healthcare to obtain a purposive sample and to ensure dependability, credibility, and transferability. Participants were of both genders and were registered nurses, nursing assistants, and care assistants. Despite including only one man, the sample was seen as representative. The constellation of participants here accurately reflects the gender distribution in municipal healthcare in general, which helps ensure credibility in the study. However, having more men to increase credibility and transferability in the study would have been desirable. The sample size of sixteen participants was seen as appropriate when performing this type of study, which strengthens the study’s credibility. Transferability is also seen as the study method and the results can be applied in other healthcare contexts.

However, some limitations must be considered when interpreting the results. One limitation could be that all participants came from the same municipality in an urban area in the western part of Sweden, thereby reducing the transferability of the results to other healthcare areas in need of care. The interview length can also be seen as a limitation; however, even short interviews included important experiences and categories were saturated in all the included interviews, again helping to ensure credibility in the study.

## Conclusions

This study contributes to the knowledge that oral healthcare is complex and cannot be achieved without establishing a relationship with the older adult who needs care. Oral healthcare comprises prerequisites such as routines, time, work-time planning, and collaboration between healthcare professionals. Registered nurses experienced an opportunity to collaborate with other healthcare professionals regarding oral healthcare and can be seen as care leaders, sharing exemplars of this in palliative care. The study also contributes to the knowledge that multi-disciplinary education programmes for healthcare professionals are appreciated and essential for improving oral healthcare.

However, future studies need to be performed to describe different groups and contexts of healthcare professionals across different cultures and languages to investigate other descriptions of healthcare professionals’ experiences of oral healthcare among older adults.

## Data Availability

The data supporting this study’s findings are available by application to the corresponding author. However, restrictions apply to the availability of these data, which were used under license for the current study and are not publicly available. Data could be made available from the corresponding author on reasonable request.
